# Intracranial epidural hematoma in a newborn with DIC secondary to congenital rubella

**DOI:** 10.11604/pamj.2015.22.63.7713

**Published:** 2015-09-23

**Authors:** Imene Dahmane Ayadi, Emira Ben Hamida

**Affiliations:** 1 Department of Neonatology, Charles Nicolle Hospital, Tunis El Manar University, Tunis, Tunisia

**Keywords:** Epidural, hematoma, congenital rubella syndrome

## Image in medicine

Epidural hematoma in newborns is rare, it occurs more frequently in infants born from nulliparous mothers with delivery difficulties. Intracranial hemorrhage in infants is usually secondary to vascular malformations, anticoagulation, inherited or acquired coagulopathy. Hematological disorders are infrequently associated with congenital rubella. The main defects of the disease associate deafness, cataracts, congenital heart disease, microcephaly and mental retardation. Rubella remains a public health problem in developing countries, in which rubella immunization is not a part of the national immunization program. We report a preterm infant, born at 31 weeks of gestation, by caesarean section for fetal distress. Prenatal ultrasound showed intrauterine growth restriction with marked oligohydramnios. The mother reported pyrexia and rush in the first missed menstrual period. Maternal rubella status was unknown. At birth, clinical examination showed microcephaly, blueberry muffin rash, diffused ecchymoses, splenomegaly, jaundice, lethargy, hypotonia and mutisite bleeding. Laboratory tests showed severe thrombocytopenia at 8 x 109/L, prolonged prothrombin time, anemia at 10.2g/dL, and hyperbilirubinemia. Urgent cranial ultrasound showed epidural hematoma with mass effect on the underlying parietal lobe and the right lateral ventricle with mid-line shift (A). It also revealed hydropcephaly sequelae of prenatal intraventricular hemorrhage; with periventricular cysts and thalamic arteritis, signs of fetopathy (B). Congenital rubella syndrome diagnosis was confirmed by a positive test of specific anti-rubella immunoglobulins M. The outcome was poor with persistence of disseminated intravascular coagulation requiring multiple fresh frozen plasma and platelet transfusion. The infant died on the third day of life secondary to refractive hemorrhage.

**Figure 1:**
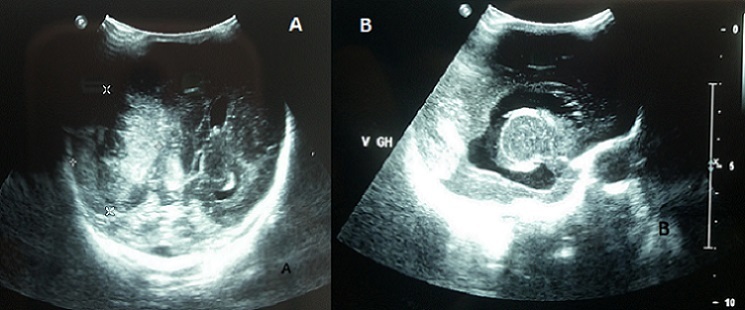
(A) epidural hematoma; (B) hydrocephaly

